# Quantitative vessel mapping on increment cores: a critical comparison of image acquisition methods

**DOI:** 10.3389/fpls.2025.1502237

**Published:** 2025-02-12

**Authors:** Richard L. Peters, Stefan Klesse, Jan Van den Bulcke, Lisa M. Y. Jourdain, Georg von Arx, Alba Anadon-Rosell, Jan Krejza, Ansgar Kahmen, Marina Fonti, Angela Luisa Prendin, Flurin Babst, Tom De Mil

**Affiliations:** ^1^ Tree Growth and Wood Physiology, TUM School of Life Sciences, Technical University of Munich, Freising, Germany; ^2^ Department of Environmental Sciences – Botany, University of Basel, Basel, Switzerland; ^3^ Forest Is Life, TERRA Teaching and Research Centre, Gembloux Agro Bio-Tech, University of Liège, Gembloux, Belgium; ^4^ Swiss Federal Institute for Forest, Snow and Landscape Research WSL, Birmensdorf, Switzerland; ^5^ Oeschger Centre for Climate Change Research, University of Bern, Bern, Switzerland; ^6^ Laboratory of Wood Technology (UGent-Woodlab), Department of Environment, Faculty of Bioscience Engineering, Ghent University, Ghent, Belgium; ^7^ UGCT - UGent Centre for X-ray Tomography, Ghent University, Ghent, Belgium; ^8^ CREAF, Catalonia, Spain; ^9^ Institute of Botany and Landscape Ecology, University of Greifswald, Greifswald, Germany; ^10^ Global Change Research Institute of the Czech Academy of Sciences (CzechGlobe), Brno, Czechia; ^11^ Department of Forest Ecology, Faculty of Forestry and Wood Technology, Mendel University in Brno, Brno, Czechia; ^12^ Department of Land, Environment, Agriculture and Forestry, University of Padua, Legnaro, PD, Italy; ^13^ School of Natural Resources and the Environment, The University of Arizona, Tucson, AZ, United States; ^14^ Laboratory of Tree-Ring Research, The University of Arizona, Tucson, AZ, United States

**Keywords:** broad-leaved species, angiosperms, quantitative wood anatomy, x-ray CT scanning, xylem porosity, uncertainty analysis, inter-and intra-annual variability, radial profile

## Abstract

**Introduction:**

Quantitative wood anatomy is critical for establishing climate reconstruction proxies, understanding tree hydraulics, and quantifying carbon allocation. Its accuracy depends upon the image acquisition methods, which allows for the identification of the number and dimensions of vessels, fibres, and tracheids within a tree ring. Angiosperm wood is analysed with a variety of different image acquisition methods, including surface pictures, wood anatomical micro-sections, or X-ray computed micro-tomography. Despite known advantages and disadvantages, the quantitative impact of method selection on wood anatomical parameters is not well understood.

**Methods:**

In this study, we present a systematic uncertainty analysis of the impact of the image acquisition method on commonly used anatomical parameters. We analysed four wood samples, representing a range of wood porosity, using surface pictures, micro-CT scans, and wood anatomical micro-sections. Inter-annual patterns were analysed and compared between methods from the five most frequently used parameters, namely mean lumen area (*MLA*), vessel density (*VD*), number of vessels (*VN*), mean hydraulic diameter (*D*_h_), and relative conductive area (*RCA*). A novel sectorial approach was applied on the wood samples to obtain intra-annual profiles of the lumen area (*A*_l_), specific theoretical hydraulic conductivity (*K*_s_), and wood density (*ρ*).

**Results:**

Our quantitative vessel mapping revealed that values obtained for hydraulic wood anatomical parameters are comparable across different methods, supporting the use of easily applicable surface picture methods for ring-porous and specific diffuse-porous tree species. While intra-annual variability is well captured by the different methods across species, wood density (*ρ*) is overestimated due to the lack of fibre lumen area detection.

**Discussion:**

Our study highlights the potential and limitations of different image acquisition methods for extracting wood anatomical parameters. Moreover, we present a standardized workflow for assessing radial tree ring profiles. These findings encourage the compilation of all studies using wood anatomical parameters and further research to refine these methods, ultimately enhancing the accuracy, replication, and spatial representation of wood anatomical studies.

## Introduction

**Figure 1:**
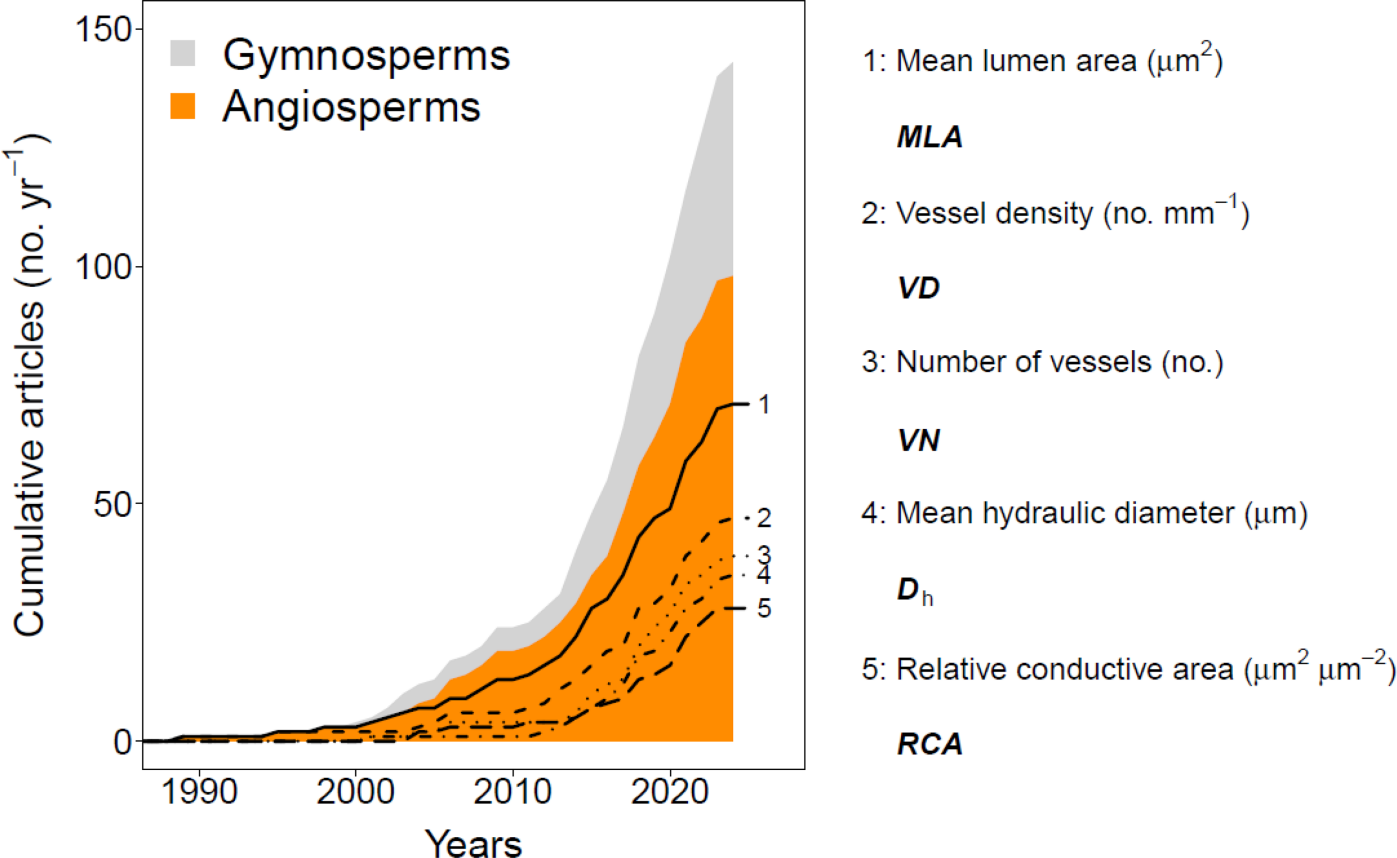
A systematic literature overview of quantitative wood anatomical studies and their use of tree-ring-specific anatomical parameters. The way this information was obtained is described in the materials and methods section. The top five main wood anatomical parameters assessed within angiosperm species are highlighted with different types of lines. Note that some studies analysed multiple wood anatomical parameters. See Materials and Methods for information on the literature review.

The anatomy of woody plants, or xylem anatomical features (i.e., xylem vessels, tracheids, and fibres, parenchyma cells and resin ducts), provides critical insights into the functioning of trees, shrubs, and herbaceous plants, as well as how they grow and interact with their environment. Wood anatomical research frequently investigates how these relationships develop over time, highlighting its significance across a diverse range of plant types (i.e., Fonti et al., 2010). More specifically, the field of quantitative wood anatomy (QWA) has made significant advancements over the past three decades. These advancements have enabled the analysis of an increasingly diverse range of xylem structural properties in trees and shrubs (**Figure 1**). High-resolution analyses of cell wall thickness and wood anatomical density patterns are now possible (e.g., Decoux et al., 2004; Rathgeber et al., 2006; Björklund et al., 2021). Key hydraulic parameters that inform water-use strategies can also be extracted (e.g., Prendin et al. 2018a; Piermattei et al., 2020; Guérin et al., 2020; Peters et al., 2023). Furthermore, QWA has provided critical inputs for mechanistic models assessing whole-tree water-use relationships and carbon allocation (e.g., Steppe and Lemeur, 2007; Peters et al., 2021; Babst et al., 2021), and sheds light on the wood formation process, highlighting both biotic (Castagneri et al., 2020b; Peters et al., 2020b; Prendin et al., 2020) and abiotic drivers in both trees and shrubs (Cuny et al., 2019). Most notably, QWA has proven more effective in reconstructing long-term climatic patterns compared to traditional tree-ring width methods (Björklund et al., 2023). All these findings underscore the value of performing QWA research. Yet, some methodological aspects of QWA practices still warrant further development and improvement.

The first QWA parameter investigated for its inter-annual variability was the lumen size of larger vessels in ring-porous broadleaves (**Figure 1**; i.e., Woodcock, 1989). This inter-annual focus progressed towards assessing the variance within a tree ring, known as intra-annual variability. The tracheidogram approach (Vaganov, 1990) marked the beginning of these intra-annual studies. This method quantifies the systematic position of conifer tracheids relative to the ring boundary, creating standardized tree-ring profiles (see Peters et al., 2018) and enabling the analysis of the impact of intra-annual climate variability on tree growth (Fonti and Babushkina, 2016; Popkova et al., 2018). Intra-annual analyses have provided more detailed climatic inferences from QWA parameters compared to ring-averaged properties (e.g., Castagneri et al., 2017a, 2018; Carrer et al., 2018). However, most studies focus on conifers (gymnosperms), while broadleaved shrubs and trees (angiosperms) are mainly assessed at the tree-ring level (e.g., Fonti et al., 2010; García-Cervigón et al., 2018; González-Muñoz et al., 2018; Anadon-Rosell et al., 2018; Nola et al., 2020). A critical step was the development of the “sectorial approach” (Castagneri et al. 2020a), which uses fixed interval widths from the ring boundary (e.g., 200 µm width bins) to calculate radial profiles of QWA parameters (e.g., Castagneri et al., 2020a; Björklund et al., 2020). Despite this method showing great potential in generating radial profiles of vessel area in angiosperms (e.g., Castagneri et al., 2020b), it has yet to be more widely applied for other QWA parameters.

Before acquiring the relevant properties of QWA parameters for inter- and intra-annual analyses, image acquisition methods must be applied to obtain digital images for analysis (e.g., von Arx and Carrer, 2014). These images enable software to detect the radial dimensions and positions of crucial transversal wood anatomical features, such as vessels and fibres or tracheid size (Peters et al., 2023). The image acquisition methods described in the literature can be categorized into three main classes: surface pictures, thin cross-sections (hence referred to as micro-sections), and micro-CT tomograms.

- Surface picture: A total of 63 out of 98 studies on angiosperms (**Figure 1**) used the surface picture approach. This method requires a clean sanded or cut surface of a wood core or disk and captures pictures of the prepared surface, often after applying contrast-enhancing coloration between the lumen and cell walls (Fonti et al., 2007; Klesse et al., 2021). The advantage of this method is its ease of use. However, the cells are not always clearly distinguishable due to poor surface preparation and limited image resolution. Therefore, this method is not suitable for samples which are smaller than a tree or a large shrub, as they have smaller cells.
- Micro-sections: A more labour-intensive method, widely applied in trees and shrubs, involves creating wood anatomical thin sections (Gärtner and Schweingruber, 2013). This method allows for high-contrast images of cell features at high resolutions (e.g., c 2 pixels µm^-^¹). However, it requires specialized pre-treatment and the wood samples need to be structurally stable, as tears and occlusions of cells can hinder accurate cell dimension detection (von Arx et al., 2016).
- Micro-CT tomograms: A non-invasive method that requires no surface preparation and keeps the wood sample fully intact is X-ray computed microtomography. This method has shown great promise in detecting wood anatomical features at various resolutions (Van den Bulcke et al., 2009, 2019; Dierickx et al., 2024). It can correct for ring and fibre alignment due to the 3D nature of the data (Van den Bulcke et al., 2014). However, it requires highly specialized equipment and is therefore less frequently used.

With the wide range of methods used in the literature and their great potential for use on angiosperms, there is a clear need for a study that verifies how comparable these image acquisition methods are for detecting QWA signals at the inter- and intra-annual scale.

The efficacy of image acquisition methods in obtaining intra-annual QWA parameters can be contextualized in a two-dimensional space. Methods range from highly invasive and labour-intensive (e.g., micro-sections) to non-invasive and low labour-intensive (e.g., micro-CT), with the second dimension including general species-specific xylem features which can impact the parameter of interest. Relevant and frequently quantified QWA parameters include the mean lumen area (**Figure 1**; Vaganov et al., 2009; Ziaco et al., 2014; Nola et al., 2020) and the hydraulic diameter of cells (*D*_h_; Lewis and Boose, 1995; Olson et al., 2014; Anadon-Rosell et al., 2018). The latter studies either looked at the climatic sensitivity of such patterns or used it to contextualize ecophysiological principles. Additionally, the theoretical xylem-specific hydraulic conductivity (*K*_s_ in m² s^-^¹ MPa^-^¹; Prendin et al., 2018a; Anadon-Rosell et al., 2022) and wood density (*ρ*; Peters et al., 2020a) are crucial for understanding water and carbon relations in trees, especially when considering the radial profile within the tree ring. Yet, in most of these studies these variables have been assessed using labour intensive micro-sections while most of these parameters can be assessed with all the above-mentioned image acquisition methods. This raises the question whether these results are comparable. The uncertainty of these methods on their respective QWA parameters must be quantified in terms of both absolute values and radial tree ring trajectories. This absolute uncertainty, along with uncertainty in the inter-annual variability, impacts the effectiveness of climate reconstructions (Büntgen et al., 2006; Ljungqvist et al., 2019). Developing an uncertainty analysis framework with these parameters and applying it across the xylem porosity spectrum (e.g., from ring- to diffuse-porous) would be highly valuable as it can help to highlight the appropriate methodology for specific QWA parameters of interest.

**Figure 2:**
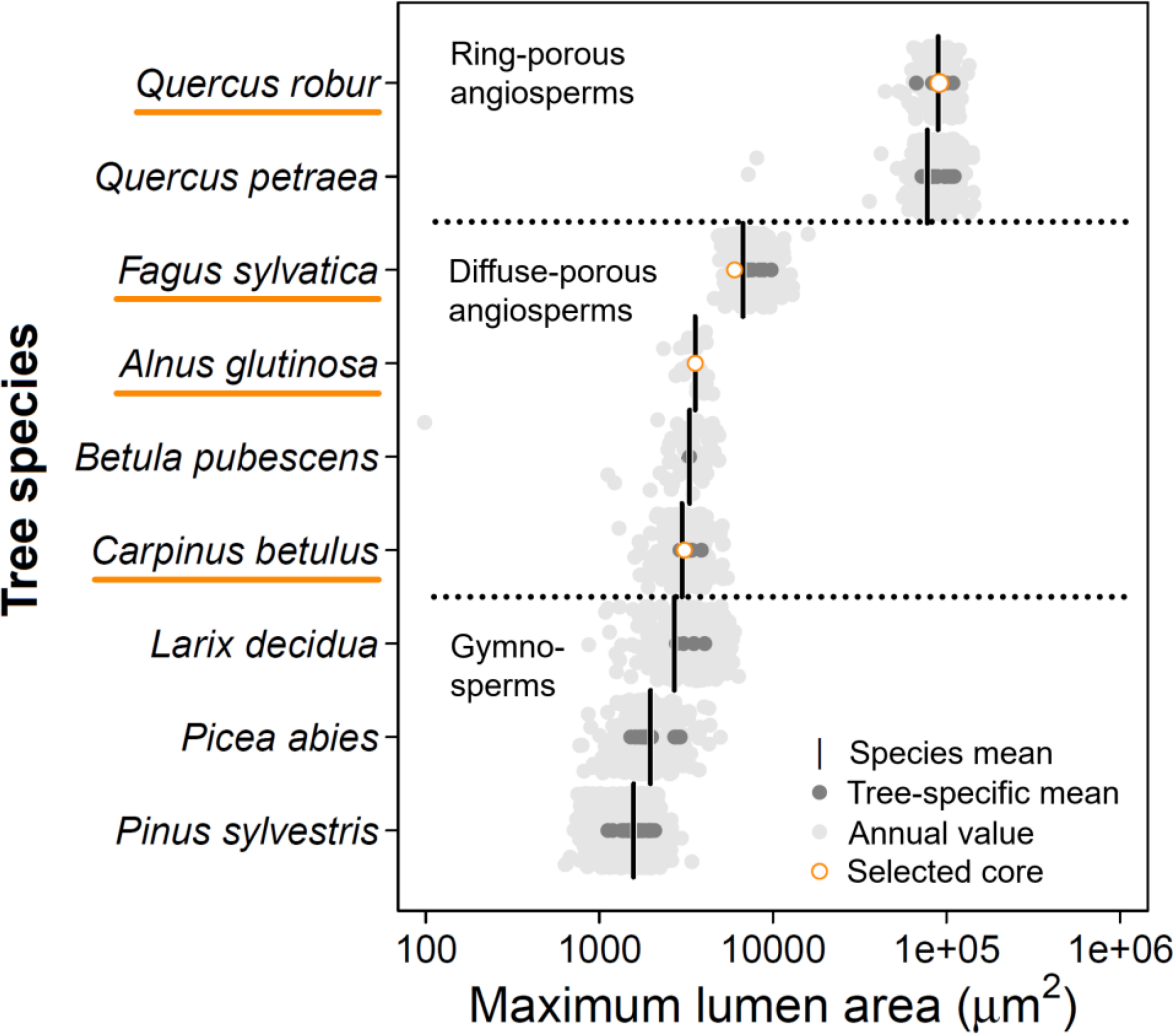
Maximum lumen area distribution across European tree species. Data was collected across the European continent (published and unpublished data; see **Supplementary Table S1**). Published and unpublished wood anatomical data were assessed and for each annual ring the maximum lumen area (99^th^ percentile for non-ring-porous species) was calculated using ROXAS. Note that the x-axis is log-transformed. The cores from the sample trees were selected along the angiosperm gradient of maximum vessel sizes (orange circles indicate the average value for the used sample). The species mean was obtained by using a mixed-effect model, where the year was nested in the individual tree as a random effect. The three conifers are added to allow comparison between angio- and gymnosperms.

In this study, we present a unique systematic uncertainty analysis of relevant inter- and intra-annual QWA parameters related to both hydraulics and carbon allocation. We performed a comparative study on a continuous range of porosity types in angiosperm tree species (**Figure 2**), including a detailed assessment of four wood samples, from diffuse-porous to ring-porous species, including *Carpinus betulus*, *Alnus glutinosa*, *Fagus sylvatica*, and *Quercus robur*. We tested the impact of the image acquisition method on the inter-annual variability of common wood anatomical parameters (as presented in **Figure 1**), but also their effect on within-tree ring radial patterns (intra-annual variability). Specifically, we developed a sectorial approach which allows us to analyse the impact of the image acquisition method on the within-tree-ring radial profile of the various parameters. For the latter, we examined *K*_s_ and *ρ*. We expect hydraulic parameters to show low absolute deviation between methods compared to carbon-related parameters. We hypothesize that hydraulic parameters depend more on larger vessels, which are easily detectable with all methods (Peters et al., 2020a). However, we do anticipate greater deviations in the inter-annual variability of QWA parameters for species with smaller maximum vessel sizes (**Figure 2**). The results of this comparative study help to advance the field of wood anatomical research into inter- and intra-annual variability of commonly used wood anatomical features.

## Materials and methods

### Literature review

We performed a systematic Web of Science literature review in May 2024 (in accordance with Foo et al., 2021) using the search terms: “Wood anatomy” and “Time series” to obtain a concrete idea on the amount of manuscripts working on QWA and which inter-annual parameters they analyse. Literature spanned from 1989 until 2024 was considered which was published in peer-reviewed English journals. Each source was inspected for relevance and afterwards stored in a database. For each stored source, we noted the assessed plant material, the location of the study, the year of publication, specifications on the sample number and annual range, the sample width (i.e., increment core width) and data treatment, the main environmental conditions driving the inter-annual variability (if reported), and the specific anatomical parameter of interest. A total of 145 studies were deemed relevant and were screened, as they dealt with wood anatomical time series, and *MLA*, *VD*, *VN*, *D*_h_ and *RCA* were selected for the inter-annual analysis within this study (**Figure 1**).

### Wood sample selection and collection

Wood samples were selected based on their maximum lumen size (in μm) and whether they cover a consistent range in the angiosperm vessel-area distribution (**Figure 2**). *Carpinus betulus* was selected at the lower end of the vessel-area distribution, while *Quercus robur* wood has the largest maximum lumen area. With this selection we also cover a large porosity gradient (i.e., from diffuse-porous to ring-porous wood). From the selected trees, one wood increment core (using an increment borer; Haglöf, Sweden) with a diameter of 5 mm and length of ≈15 cm was taken (**Table 1**). During the selection we ensured that there is no clear signs of tension or reaction wood. Sample selection was based on the quality of the sample (i.e., no cracks or rotten sections), covering at least 10 years of annually distinct rings. With these criteria a collection of angiosperm wood samples was obtained where for each core the three image acquisition methods were applied (**Table 1**). For each of these samples QWA analysis was performed using the three specified image acquisition methods, surface pictures, micro-sections, and micro-CT volumes. Due to the nature of the methods, it is impossible to make images of exactly the same wood surface area due to vertical offsets.

**Table 1:** Characteristics of trees from which wood samples were obtained and analysed with the three methods.

Site (Country)	Latitude (°N)	Longitude (°E)	Species	Tree (Core label)	*d*_stem_ (cm)	*h*_plant_ (m)	*h*_sample_ (m)	*A*_fibre_ (μm^2^)	*t*_range_ (yr)	*w*_bin_ (μm)	Source
Lanžhot, floodplain forest (CZ)	48.68148	16.94647	*Quercus robur*	QURO108	60.3	35.5	1.3	500	2006-2018	450	Kowalska et al., 2020
Soroe, ICOS flux tower site (DK)	55.48694	11.64583	*Fagus sylvatica*	FASY24	55.5	30.7	9	500	1995-2017	120	Peters et al., 2020a
Wöpkendorf, peatland forests (DE)	54.13471	12.51273	*Alnus glutinosa*	ALGL01	38.4	23.4	1.3	500	1989-2017	120	Anadon-Rosell et al., 2022
Hofstetten, Basel University crane site (CH)	47.46864	7.50235	*Carpinus betulus*	CABE4	34.6	24.6	1.3	500	1960-2018	120	Dietrich et al., 2018

For each individual tree, diameter at breast height (*d*_stem_), plant height (*h*_plant_), and the height at which the sample was taken (*h*_sample_), were measured. For the sectorial processing (described below), fibres were distinguished using a specific size threshold (*A*_fibres_). The annual range of rings within the sample is also provided (*t*_range_). The width of the bins utilized within the sectorial approach is variable for each sample (*w*_bin_). The source column provides the manuscript that described the sampling site and design.

### Surface picture

The wood surface of each core was prepared by using a core microtome (Gärtner et al., 2015) to create a level transversal surface. The wood surface was blackened using a black marker and the cell lumina were filled by carefully rubbing white chalk powder onto the sample to enhance the contrast between cell wall and lumen (i.e., Fonti et al., 2007; Klesse et al., 2021). The prepared samples were positioned within an adjusted Atrics system (Levanič, 2007), referred to as “Skippy”, consisting of a motorized stage (minimum precision 0.01 mm) with a controlling unit used for moving the camera from the bark to pith direction (Gärtner et al., 2024). A high-resolution Canon 5DsR camera with a 100 mm macro lens (Canon Inc. Tokio, Japan) was used to obtain surface pictures. Images along the radial direction were stitched using PTGui (New House Internet Services B.V., Rotterdam, NL; see von Arx et al., 2016). The camera settings were set to an f-value of 7.2, with an obtained theoretical maximum resolution of 4.14 μm (or 0.2381 pixels per μm, 6,148 dpi) defined by the camera’s image sensor and the true 1:1 image reproduction ratio of the macro lens. Calibration values were generated by performing multiple measurements of mm-paper for each image. On average our pictures have a resolution of 6000 ± 25 dpi (based on laboratory-tested user-based in-accuracy during image resolution calibration). Stitched images of the entire sample were exported as JPEG files usable within image analysis software. Due to the difficulty in identifying tree rings on a species like *C. betulus*, we were unable to use the surface picture of this species within the comparison.

### Micro-section

Classical wood anatomical micro-sections were taken from the transversal surface (Gärtner and Schweingruber, 2013). Wood samples were split into ≈4 cm radial samples using a cutter and prepared for paraffin embedding, to stabilize the tissue for obtaining high quality thin sections (von Arx et al., 2016; but see Frigo et al., 2024). Almost all samples, except the samples from *A. glutinosa* (see Anadon-Rosell et al., 2022 for details on processing), were embedded in paraffin according to standard protocol using a tissue processor (Leica TP1020, Leica Biosystems, Nussloch, Germany; detailed in Hamann et al., 2011). Each paraffin block was placed within a rotatory microtome (Leica RM2245, Leica Biosystems, Nussloch, Germany) to cut 12 μm thick cross-sections. The thin sections were stained with 1:1 safranin and astrablue solution and permanently fixed on glass slides using Euparal. Digital images of the sections with a resolution of 0.44 μm (or 2.27 pixel per μm, 57,600 dpi) were taken using a slide scanner (Axio Scan Z1, Carl Zeiss AG, Germany). Automatically stitched images were exported as JPEG files for further image analysis.

### Micro-CT X-ray scanning

Each wood sample was measured for transverse wood anatomical features along the radial direction using the Nanowood X-ray CT scanner. This scanner was custom-built at the UGent Centre for X-ray Tomography (UGCT, www.ugct.ugent.be; Ghent, Belgium) and recently refurbished in collaboration with TESCAN-XRE (TESCAN ORSAY HOLDINGS a.s.), a former UGCT spin-off company. It is specifically designed to study materials made of wood or derived from wood. The X-ray source, a nano focus tube, was used to scan the samples at an average voltage of 50kV and a current of 40 μA, using 700 ms exposure time per projection and 3600 projections. All scans in this study were reconstructed using the Octopus Reconstruction software (Vlassenbroeck et al., 2007). With the described setup, the obtained reconstructed voxel pitch, further referred to as resolution, was 3.6 × 3.6 × 3.6 μm, which is similar to the 0.277778 pixel per μm and was used for the comparison between the image acquisition methods (7,060 dpi). Although submicron resolutions are obtainable with this system (Van den Bulcke et al., 2019), we optimized the resolution for both comparability and scanning time-efficiency. Scanning was performed along the entire wood sample length. A “slice” was selected parallel and closest to the surface, to maintain comparable anatomical features compared to the other methods (i.e., reducing variability in both cell size and position when moving away from the surface). The slice was pre-processed to remove noise and enhance the image, by applying histogram equalization to transform the values of the grey-scale image such that contrast improved. Subsequently the images were binarized using the topological derivative of Larrabide et al. (2008). All image processing was performed using MATLAB^®^ and Octopus Analysis+ (Vlassenbroeck et al., 2007), to binarize the image and de-speckle the image, and export the image into the JPEG format for further analysis.

### Image analysis with ROXAS

The specialized software ROXAS (von Arx and Carrer, 2014), based on Image-Pro Plus (Media Cybernetics, Rocksville, MD, USA), was used to semi-automatically measure the lumen size of conductive cells for each method (fibres and vessels in broad-leaved species; von Arx et al., 2016). Each imaging method was analysed with their specified resolution (in pixel per μm), while using a species-specific ROXAS configuration file (see von Arx and Carrer, 2014). Selecting the exact same area for analysing the wood was not possible, as the analysed surface was not at the same sample depth between methods, moreover some methods allow for a wider area of interest which we deem part of the merits of a specific method. For all methods, care was taken to manually detect any previously undetected vessels within the section. Here, we assumed high sample quality for our comparison. Moreover, for the thin section with the highest resolution, detailed manual corrections were performed to draw most fibre lumina which serve as the true reference for comparison. A general distinction between fibres and vessels was used where elements with a lumen area above 500 μm^2^ were considered as vessels (see **Table 1**; **Supplementary Figure S3**). This threshold was selected based on manual observations and the fact that we wanted a uniform threshold across species, instead of smaller species-specific thresholds (see Peters et al., 2020a). Ring boundaries were manually drawn and this information was stored for informing the inter-annual and sectorial approaches, described below. For each detected cell, we recorded: 1) the year in which the cell is positioned, 2) the calibrated positional information (*X*_cal_ and *Y*_cal_ in μm within the annual ring), 3) the lumen area (*A*_l_ in μm), and 4) the radial distance from the ring boundary (RadDistR in μm and % of the annual ring).

### Inter-annual comparative analyses

To investigate the results of the different imaging methods to capture ring-specific QWA parameters at the tree-ring scale, we calculated for each ring the top five most commonly used parameters (**Figure 1**): Mean Lumen Area (*MLA*), Vessel Density (*VD*), Vessel Number (*VN*), Mean hydraulic diameter (*D*_h_), and Relative Conductive Area (*RCA*). As the different imaging methods will detect different minimum sized cells, we performed a threshold analysis across all rings where we assessed when the *MLA* of the average tree rings converged between the different methods (**Supplementary Figure S1**). Specifically, within this analysis we inspected at which cut-off value the mean lumen area is different between the methods, which is caused by the difference in the methods capability to detect smaller xylem elements. For the diffuse porous species (*C. betulus*, *A. glutinosa*, and *F. sylvatica*) the cut-off was set at 1000 μm^2^, while 5000 μm^2^ was used for the ring-porous species (*Q. robur*). These thresholds are commonly used thresholds in literature (i.e., Granda et al., 2018; Klesse et al., 2021). For each ring the cell properties and the total number of visible features were obtained from ROXAS, where *VD* and *RCA* were calculated using the area of interest, measured manually for each ring. *VN* was calculated with the *VD*, by multiplying this number by the ring area which was calculated using a fixed 5 mm width of the core surface and the ring width measurement. This was done to avoid that wider ring areas would bias *VN*. For each annual ring we also considered xylem anatomical parameters which relate to hydraulic efficiency. The key parameter describing this efficiency in literature is the hydraulically weighted mean vessel diameter (*D*_h_) calculated as described in Equation 1 (Kolb and Sperry, 1999). Thereby, the cell-specific hydraulic diameter (*D*_h.cell_) was used to account for elliptical lumen areas (Lewis and Boose, 1995).


(1) $$D_{\text{h}}=\;\frac{\sum^{\;}D_{\text{h}.\text{cell}}5^{\;}}{\sum^{\;}D_{\text{h}.\text{cell}}4^{\;}}$$

### Intra-annual comparative analyses

To see the effect of the method on the intra-annual tree-ring variability, we performed a sectorial approach, generating tracheidograms for the samples based on distance instead of on cell count (Peters et al., 2018), and determined ecophysiologically relevant parameters such as hydraulic conductance and wood density. We used the positional information obtained from the vessels and fibres (*X*_cal_ and *Y*_cal_ of the cell centroid) in combination with the ring boundaries. Each sector was assigned a fixed radial width (from the pith to the bark direction) for which a “sector box” was determined. For all species, except for *Q. robur*, we used a fixed sector width of 120 μm which corresponds to the average diameter of larger vessels in these species. For *Q. robur* we selected a larger sector width of 450 μm, as the earlywood vessels are substantially larger (**Table 1**). After isolating all cell elements, with their centroid in the sector box, three QWA features were extracted and linked to the central radial distance of the sector box. First, the median lumen area was determined, as initial testing confirmed that this was more robust than using the *MLA*. Second, the theoretical hydraulic conductance (*K*_H_ in m^4^ MPa^-1^ s^-1^; see Equation 2) was determined as a useful hydraulic proxy to define rehydration potential (Peters et al., 2023), which relates to the *D*_h_. *K*_H_ was calculated according to the Hagen-Poiseuille law (Prendin et al., 2018b; Tyree and Zimmermann, 2002), where the conductance of a capillary tube is defined by its length (*l*) and lumen diameter (*d*_lum_), with *η* being the dynamics viscosity of water (0.001 Pa S at 20°C). *K*_H_ of the sectors was then divided by the area of the sector box to obtain the specific theoretical hydraulic conductivity (*K*_s_; m^2^ s^-1^ MPa^-1^), which is independent from the size of the sector. Third, wood density (*ρ* in g cm^-3^) of each sector was determined as detailed in Peters et al. (2020a) where the optical density, of the total non-lumen area divided by sector box area, was calculated to obtain *ρ* by assuming a fixed density of wall material of 1.504 g cm^-3^ (Kellogg and Wangaard, 1969). Specifically, for the thin-section images, we generated two radial profiles of these QWA parameters, 1) isolating vessels only, and 2) including vessels, fibres and estimating the number of undetected fibres according to Peters et al. (2020a). The latter was applied to get an estimation of the absolute values of the parameters when being able to detect all non-vessel structures (excluding cracks), while accounting for cracks within the sample and assuming a fixed cell wall thickness of 4 μm to determine the total area covered by cells against the sector box area (see https://deep-tools.netlify.app/2020/11/24/sector-intro/; see **Supplementary Figure S2** as an example). See Peters et al. (2020a) for a more detailed description on the sectorial approach.


(2) $$K_{\text{h}}=\;\frac{\pi \sum^{\;}d_{\text{lum}}4^{\;}}{128\eta l}$$

### Uncertainty analyses

All data analyses were performed using the R software (version 4.0.2, R Core Team, 2017), including the inter-annual uncertainty analysis and the sectorial approach. For this analysis we chose the wood anatomical thin section as a reference, due to its high resolution, and time spent annotating in ROXAS. For the wood anatomical thin section (or WA) we calculated all parameters once with and once without the cut-off, whereas for the other two methods we calculated the parameters considering the cut-off value. The other methods were only considered using the cut-off value for the cell-specific lumen area. Linear regression analyses were performed between the annual time series of *MLA*, *VD*, *VN*, *D*_h_, and *RCA*, quantifying the goodness of fit (*R* ^2^), providing information on the matching inter-annual variability, the linear regression slope between the image acquisition methods, providing information on the slope, and the relative offset to the 1:1 line (in %), to show the over- or underestimation of the method compared to the reference. Significant relationships (*P* < 0.05) were reported where relevant.

We moved beyond inter-annual analysis by assessing the within tree-ring patterns of wood anatomical parameters which are relevant for ecophysiology, more specifically *K*_s_ and *ρ*. We calculated the mean time-series of these parameters across sectors for each year and aggregated them to describe the general radial profile per species and method. To describe the general behaviour of *K*_s_ and *ρ* along the tree-ring across years, we used a generalized additive mixed model (gamm). The gamm model was fitted using the “mgcv” package (Wood, 2017), while considering method as a fixed effect and the year as a random intercept. For each fit we calculated the standard error and subsequently the upper and lower confidence interval. Significant differences (*P* < 0.05) were reported in comparison with the wood anatomical thin section which included the cut-off threshold.

## Results

### Cell size and frequency effects

**Figure 3:**
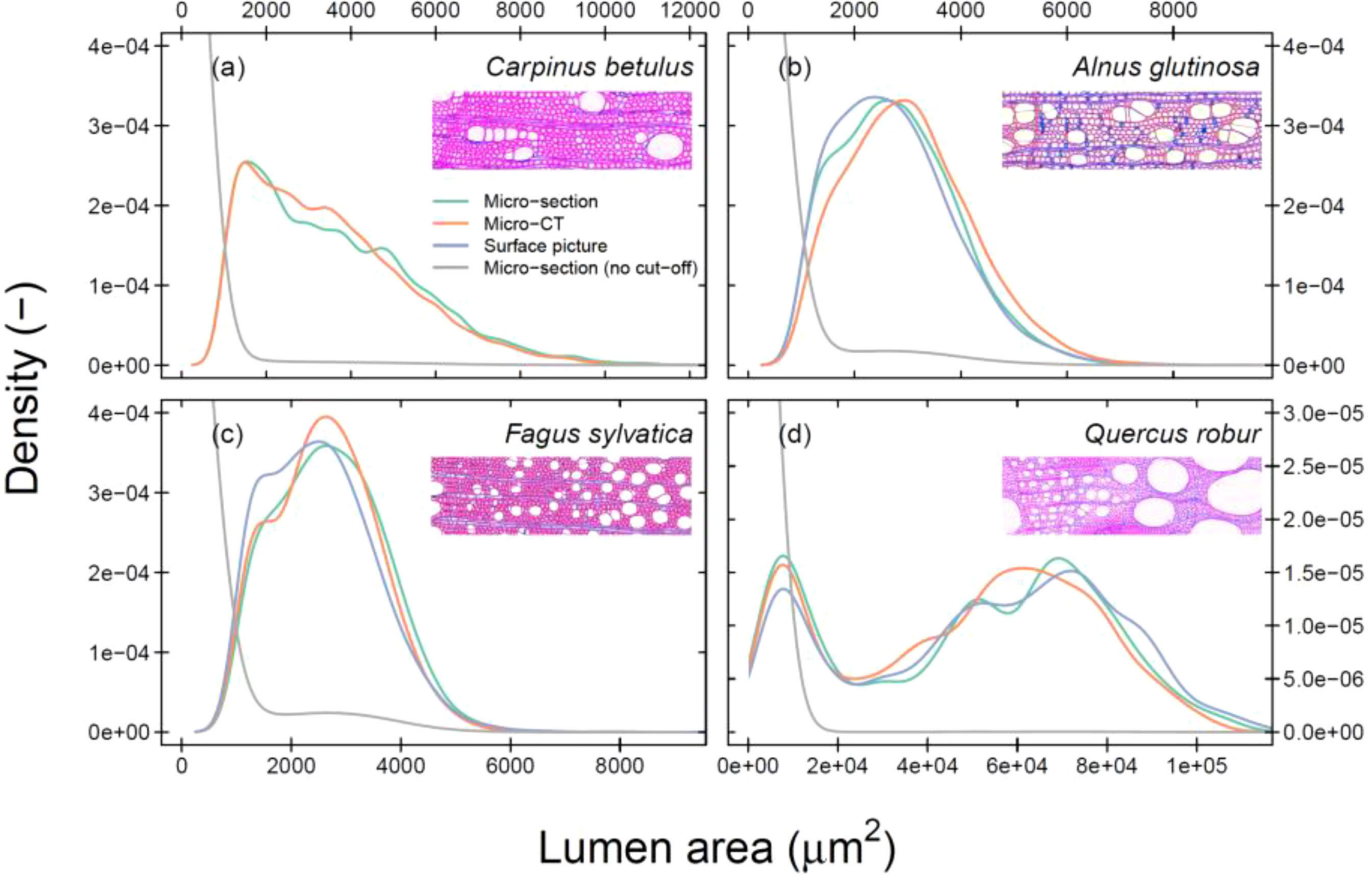
Density plot of the cell-specific lumen area distribution across all tree rings per species. Cut-off data is presented to highlight the variance across larger cells (see **Table 1** for used cut-off values). The colours indicate the image acquisition methods. The grey-line shows the raw data of a wood anatomical thin section. The unscaled images illustrate the specific wood structure for the species *Carpinus betulus* **(A)**, *Alnus glutinosa* **(B)**, *Fagus sylvatica* **(C)**, and *Quercus robur* **(D)**. Mind that there is no surface picture for *C. betulus*. Mind that the x-axis of each panel does not have the same extent.

We found strong similarities between ring-width measurements obtained from different image acquisition methods (*P* < 0.001; **Supplementary Figure S4**), proving that the image calibration was appropriate and the differences between methods should be mainly caused by variability in the cell size, image resolution, and subsequent detection. When assessing the frequency distribution of cell-specific lumen area across all tree rings, the general shape of the relationship is similar across methods (**Figure 3**). *C. betulus* shows a consistent skewness towards lower vessel lumen area, while *Q. robur* shows a bimodal distribution with smaller latewood vessels and large earlywood vessels. These findings highlight that across years the general distribution of cell lumen area is well captured by all methods when applying a consistent cut-off value (see **Supplementary Figure S5** for results without a cut-off). When analysing the detection of specific cell sizes, it was clear that the micro-CT and surface picture techniques (in their current setting) are not capable of detecting most fibres, in contrast to wood anatomical thin sections (**Supplementary Figure S5**).

### Matching inter-annual variability

**Figure 4:**
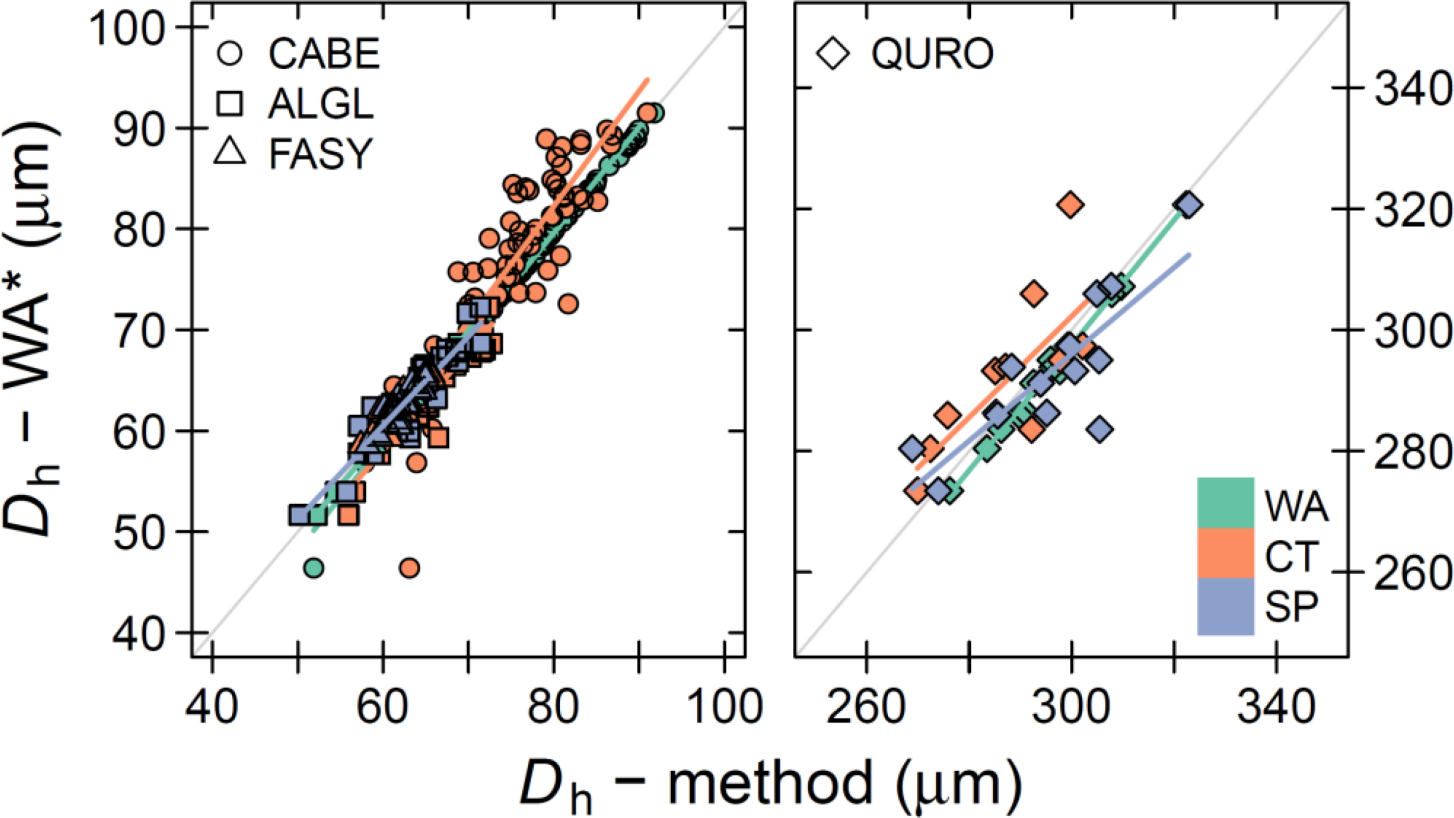
Relationship between the mean hydraulic diameter (*D*_h_) across image acquisition methods. The annual *D*_h_ measurements from the wood anatomical (WA) thin section that considers fibres and vessels (*D*_h_ – WA*) is compared to the annual *D*_h_ measurements determined with the specific image acquisition method (*D*_h_ – method). The bold coloured line shows the linear relationship between the methods. Mind that for the x-axis values, a species-specific threshold was used to remove smaller cells (see **Table 1**). The methods presented on the x-axis are treated with the size cut-off threshold. Colour indicate the different methods: WA = thin section, CT = Micro-CT X-ray tomography, and SP = surface picture. The symbols highlight the different species: CABE = *C. betulus*, ALGL = *A. glutinosa*, FASY = *F. sylvatica*, QURO = *Q. robur.* The grey line shows the 1:1 line.

We now focussed on the inter-annual tree-ring variability, with a special emphasis on the hydraulic parameters (i.e., *D*_h_). The inter-annual variability in *D*_h_ was well captured, when comparing the raw wood anatomical thin section (including fibres and vessels) to the other methods (treated with the cut-off; **Figure 4**). Here we used all wood anatomical elements (both fibres and vessels) as a reference to highlight the robustness of the parameter, however in analysing the other parameters we used the WA parameters calculated when using a cut-off. All species considered, we found no significant effect of the method on the linear relationship (*C. betulus, P > 0.45; A. glutinosa, P > 0.20; F. sylvatica, P > 0.10, and Q. robur P > 0.33*). The highly significant slopes (*P* < 0.0001) between the linear relationships ranged from 0.7 to 1.1, with the weakest relationships apparent for *Q. robur* (average slope of 0.8 for all methods), likely caused the relatively low amount of detect vessels and its variance between methods. The average goodness of fit (*R* ^2^) across species was 0.83, with an average offset for the residuals to the linear model line of around -1.1 μm.

**Figure 5:**
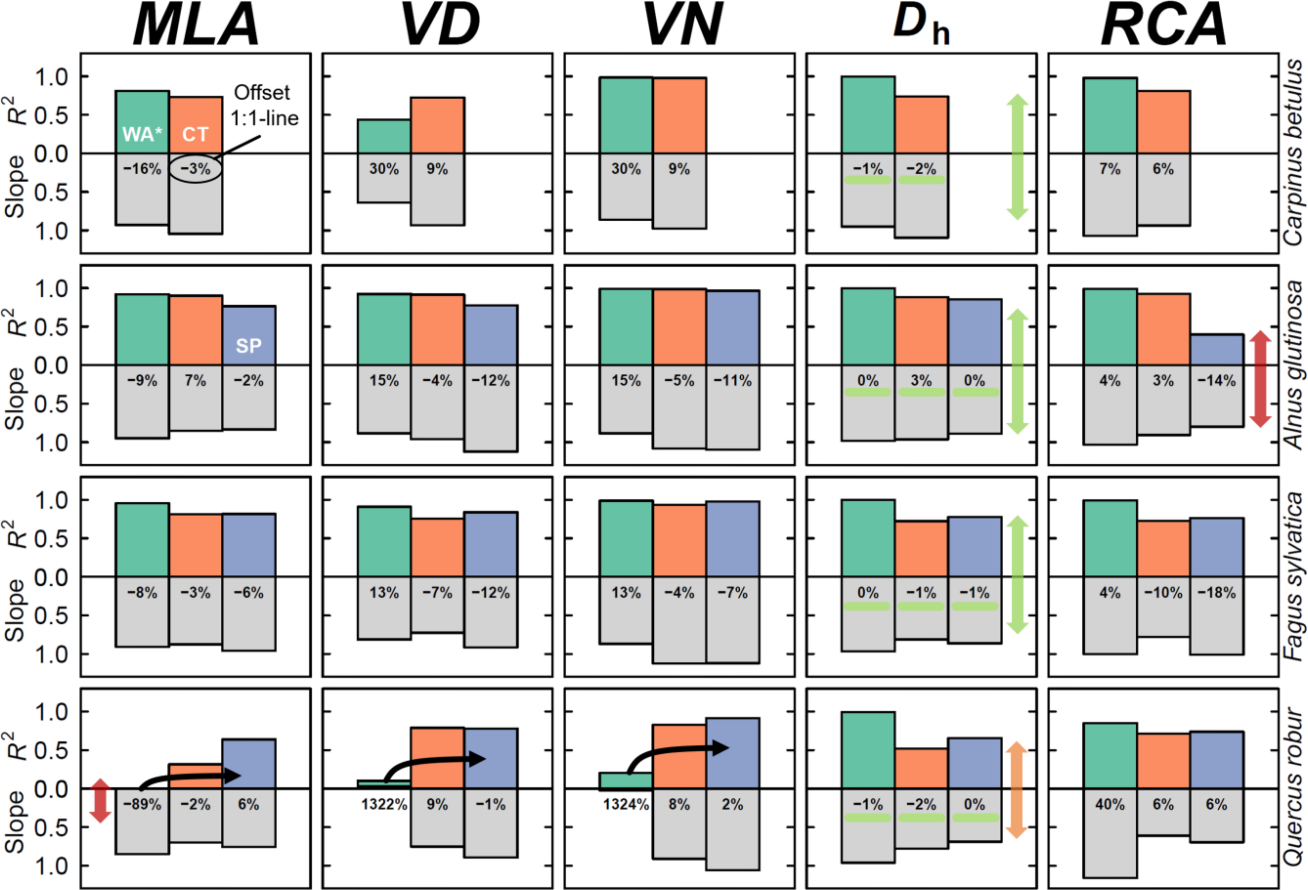
Common inter-annual variability among methods and species. The output of simple linear regressions among methods, including the goodness of fit (*R* ^2^; upward coloured bars), the regression slope (downward grey bars), and the mean residual offset in % from the 1:1 line (shown in the grey bars). The closer both bars are to 1, the better the similarity is between both the inter-annual variability and the directionality of the linear fit. Green arrows and highlights indicate well performing parameters, whereas orange and red arrows indicate lower performing parameters. Black lines for *Quercus robur* highlight the trajectory of the relationship. All methods are compared to the wood anatomical thin section method where a cut-off threshold was applied. Methods include; WA* (green) = thin section including vessels and fibres, CT (orange) = Micro-CT X-ray tomography, and SP (blue) = surface picture.

For the five most commonly analysed wood anatomical parameters (**Figure 1**), we compared all methods to the wood anatomical thin-section to which we applied a cut-off (**Figure 5**). For *D*_h_, it is clear that both the *R* ^2^ and slope are high when comparing the micro-CT and the surface pictures to the wood anatomy on which a cut-off was applied (on average 0.73 and 0.86, respectively). Especially, the deviation from the 1:1 line was exceptionally small for this parameter (ranging from -2.5% to 2.9%). For this parameter the inter-annual variability was best described when comparing the raw thin section (including vessels and fibres) with the cut-off thin section (mean across species; *R* ^2^ = 0.99, slope = 0.96, residual offset = -0.91). Again, the weakest common pattern in inter-annual *D*_h_ was found for *Q. robur*. For the diffuse-porous species, the most commonly used *MLA* also appeared to be well captured by all methods, even when including fibres (**Figure 5**; mean *R* ^2^ = 0.84, slope = 0.92, residual offset = -160 μm^2^, or -1%). For these species it was interesting to see that even when including fibres, which caused a mean residual offset of -342 μm^2^, the inter-annual variability was well maintained (mean *R* ^2^ = 0.90). *Q. robur* showed more discrepancy between the methods, where there was poorly explained inter-annual relationship of *MLA* when considering fibres within the thin section (R^2^ < 0.01). When considering the micro-CT and surface picture this inter-annual commonality with the thin section improved slightly (mean *R* ^2^ = 0.48, slope = 0.73), although it was still the lowest value among the species (see **Figure 5**).

Wood anatomical parameters related to the frequency of detected vessels were more variable in their performance, compared to cell-size related parameters such as *MLA* or *D*_h_. Particularly the *VD* showed a low *R*_2_ of 0.58 for *C. betulus* with a highly variable slope range across species (min = 0.64, max = 0.93; **Figure 5**). The *VN* parameter (which was calculated based on *VD* and the ring area) showed better inter-annual comparability for *C. betulus* and *A. glutinosa*, and *F. sylvatica*, but less for *Q. robur* (**Figure 5**). The *RCA* is also impacted by the image acquisition method, with a reduced common inter-annual variability when considering the surface picture for *A. glutinosa* (*R* ^2^ = 0.40), which underestimated the *RCA* by 14%. However, *Q. robur* showed a high inter-annual agreement for both *VD*, *VN*, and *RCA*, although when including the fibres particularly the inter-annual variability be more consistent for *VD* and *VN* compared to the other species.

### Sectors and the intra-annual variability

**Figure 6:**
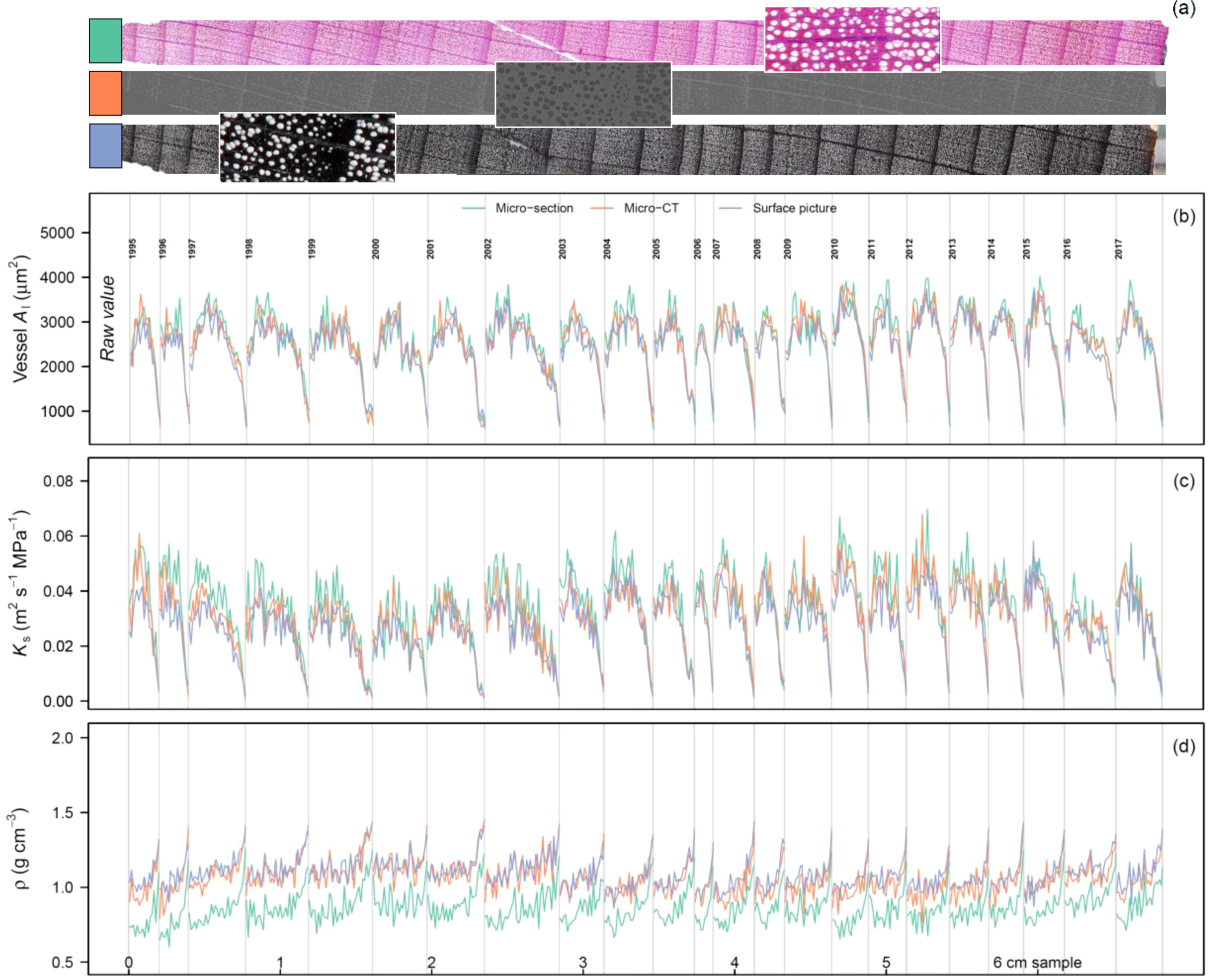
Intra-annual time-series for *Fagus sylvatica* based on the sectorial approach. Along the tree core **(A)**, 120 μm long sector boxes were used to calculate the median lumen area of the vessels (*A*_l_; **B**), the specific theoretical hydraulic conductivity (*K*_s_; **C**), and the sector density (*ρ*; **D**). Each grey line highlights a ring-width boundary. Each of the coloured line shows a different image acquisition method: a wood anatomical thin-section **(A)**, micro-CT, and surface picture.

**Figure 7:**
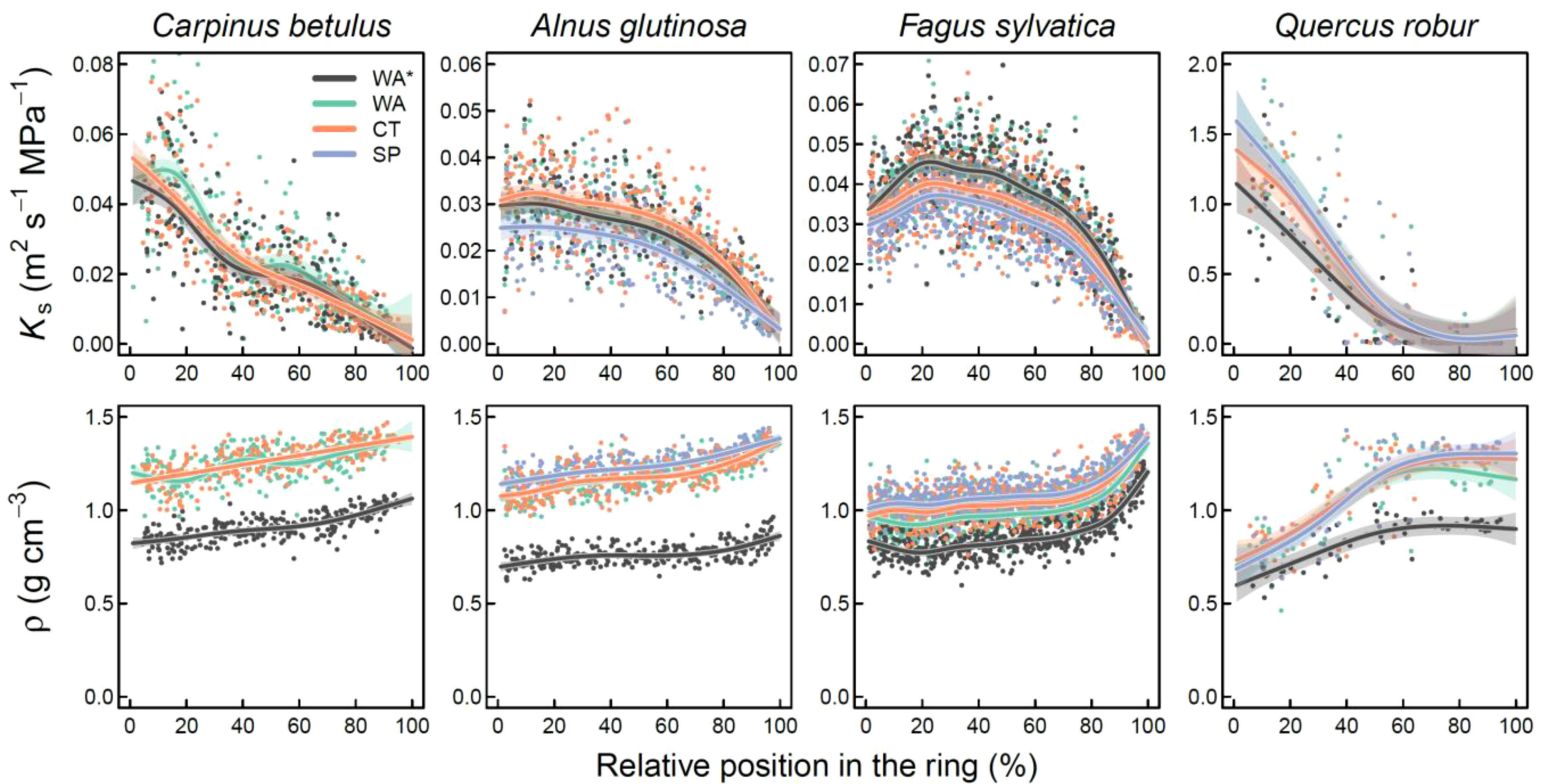
Radial profile of the specific theoretical hydraulic conductivity (*K*_s_), and the sector density (*ρ*) across years. The generalized additive mixed-effect model (gamm) fitted mean and the confidence interval for each method is provided in their respective colour. Methods include wood anatomical thin sections which include fibre lumina measurements (WA*), wood anatomical thin section with a cut-off value for all cells (WA), Micro-CT (CT), and a surface picture (SP). Mind that in this graph all sectors were considered relative to their respective annual tree ring width (or relative position in the ring in %). Mind that in some plots the green curve is not visible due to overplotting.

For each year the radial pattern matched well among the different species (i.e., **Figure 6** for *F. sylvatica*). *A*_l_ and *K*_s_ showed highly similar patterns and as such we only considered *K*_s_ for further analyses. When considering the general radial profile across years, it is clear that all methods showed highly similar *K*_s_ trends (**Figure 7**). Slight differences can be found for the surface picture when considering *A. glutinosa* and *F. sylvatica* where *K*_s_ is slightly underestimated at the beginning of the tree ring (*P* < 0.001). This is however not the case for *Q. robur* (*P* = 0.955), where only *K*_s_ is significantly reduced in the first few sectors when the fibres are considered (*P* < 0.001). For the latter the variability in *K*_s_ in the latewood cannot be captured with the micro-CT or surface picture method, visible as low values close to zero (**Figure 7**). For *ρ*, across all methods it is clear that the absolute density is lower when considering the fibre lumina compared to solely considering the vessels (**Figure 7**). This effect is less apparent in a species like *F. sylvatica*, where we do see an increase in density in the latewood. For *Q. robur* the density dynamics are also different when considering fibres with a more gradual increase in *ρ* compared to when considering only the larger vessels.

## Discussion

In this study we performed a systematic uncertainty analysis on the three most commonly applied image acquisition methods used in the field of quantitative wood anatomy (QWA). Overall, we found inter-annual agreement between the methods when assessing the most frequently used tree-ring specific wood anatomical parameters. Cell-size related parameters were most accurately captured across methods, compared to those which are more strongly dependent on vessel numbers (**Figure 5**). We also presented a standardized work flow in the R programming language with which radial profiles for angiosperms can be established (equivalent to the tracheidogram approach; Peters et al., 2018). This approach revealed that although wood density is overestimated in most methods, due to the simplistic assumption that everything that is not a vessel is cell wall, radial profiles of hydraulic parameters are well described across methods. Moreover, the sectorial approach (**Figure 6**) could expand the use of the highly promising intra-annual variability for wood anatomical features of angiosperms (i.e., Castagneri et al., 2020b).

### Robust hydraulic parameters across methods

Our inter-annual analyses of common wood anatomical parameters revealed that the mean hydraulic diameter (*D*_h_) matched well among methods (**Figure 4**). This is likely due to the overall common cell size distribution and the appropriate size estimation of the largest cells for this parameter across the three methods (**Figure 3**). Both the goodness of fit (*R* ^2^) and slope of the regression between methods were high. Most notable was the strong mean prediction accuracy (the offset from the 1:1 line), which confirms that studies using either of these methods can be compared for their climate-*D*_h_ relationships (i.e., Nolin et al., 2021; Castagneri et al., 2017b). Moreover, the fact that *D*_h_ is not affected by the in- or exclusion of fibres (**Figure 4**) displays that this parameter is robust and not strongly depending on a size cut-off commonly used in ring-specific analyses (i.e., Fonti and García-González, 2004; Akhmetzyanov et al., 2019). This is further illustrated by the accurate capturing of the within ring variability of the specific theoretical hydraulic conductivity (*K*_s_) across methods (**Figure 7**), paving the way for future analyses on the specific sectors and their climatic sensitivity (Castagneri et al., 2020a).

Ring-porous *Q. robur* showed the lowest *R* ^2^, especially when comparing *D*_h_ variability among image acquisition methods (**Figure 5**). One explanation could be the relatively lower number of vessels per analysed surface area and the fact that we do not measure exactly on the same location and extent within the wood. This highlights an important limitation with ring-porous species, as one would require the inclusion of more cells to get more robust signals. While for *F. sylvatica* it was found that a QWA signal is relatively stable for a 1 mm wide radial strip (Diaconu et al., 2017), for ring-porous species like *Q. robur* or *Fraxinus excelsior* this might require a larger sampling width (García-González and Fonti, 2008). This impact is also presented within the most widely used parameter for ring-porous species, the mean lumen area (*MLA*), indicating the need for a more detailed assessment on the sampling width impact across wood anatomical parameters. Nevertheless, the robustness of these hydraulic parameters is highly promising as they describe the efficiency of water transport though the xylem tissue of the plant (Tyree and Zimmermann, 2002) and have more recently been linked to the rehydration efficiency of tree species after drought (Peters et al., 2023).

### Evaluation of commonly applied image acquisition methods

The most common applied image acquisition method from the literature review for angiosperms is the surface picture method, followed by the wood anatomical thin section method. From the inter-annual analysis, it is clear that with surface pictures at 6000 dpi most variability is well captured, as long as the cells are not too small (see *A. glutinosa* in **Figure 5**; maximum lumen area < 4000 μm^2^) or the ring-boundaries are properly visible (i.e., not the case for *C. betulus*). On the intra-annual level, the surface picture method performs well in capturing the decrease in *K*_s_ across rings (**Figure 7**). However, the wood anatomically derived density (*ρ*) is clearly overestimated compared to the reference (a thin section which includes vessels and fibres) due to the fact that this method is unable to detect the lumen of the fibres (as discussed by Peters et al., 2020a). This is related to the general downside of this method, which is the time-consuming preparation of the surface, using for example a core microtome (Gärtner and Nievergelt, 2010). This forces the practise of sub-selecting wood samples due to the wood quality issues which do not provide the appropriate contrast (i.e., Skiadaresis et al., 2021). Moreover, for surface pictures there is a technological resolution limit (defined by the resolution of the camera sensor and magnification of the lens), which hampers the use of higher resolution images to detect wood anatomical structures. This is specifically illustrated by the relatively high underestimation of *VN* and *RCA* for the surface picture method for a species like *A. glutinosa* (**Figure 5**). This resolution issue is also important for ring-porous species, as the *MLA* is highly sensitive to different size cut-off values (**Supplementary Figure S1**). Nevertheless, the equipment requirements of this method are low and sample processing is fast, which explains its common use for ring-porous species (St. George et al., 2002; Fonti et al., 2009; Klesse et al., 2021). Moreover, the method can be applied on larger surface areas, such as parts of a stem disk, alleviating some of the issues due to limiting amounts of vessels captured with a 5 mm wide core (García-González and Fonti, 2008; Fonti et al., 2009). Expanding the use of this method to (semi-)diffuse porous species like *F. sylvatica* would thus be recommended.

Wood anatomical thin cross-sections show great common patterns with the other methods and allow for the disentanglement of the impact of vessels and fibres on the wood anatomical parameters (**Figures 5**, **7**). However, the creation of wood anatomical micro-sections requires a multitude of processing steps and specialized equipment (von Arx et al., 2016; Fonti et al., 2025). Moreover, not all wood material is easily processed due to either the brittle nature of material (Reinig et al., 2018) or the hardness of the wood sample. Despite these challenges, thin sections are the ideal standard to measure wood anatomical features due to the contrast enhancing staining and high optical resolution (i.e., 2.27 pixel per μm; see also Gärtner and Schweingruber, 2013). Moreover, due to the easy detection of fibre lumina at the ring boundary, ring detection was not difficult for *C. betulus* when using this method (**Figure 3**). Furthermore, this method is most commonly used for gymnosperms and shrubs more generally (i.e., Buras et al., 2017; Seftigen et al., 2022) and shows great potential in extracting wood anatomically derived density (**Figure 7**; Peters et al., 2020a). Expanding the use of this method to other angiosperms, in combination with the sectorial approach, would provide a novel milestone for exploring the use of intra-annual variability in wood anatomical parameters as climate reconstruction proxies.

### Potential for micro-CT x-ray tomography

The X-ray microtomography is the least applied method in QWA literature, yet shows great potential in reconstructing the inter- and intra-annual variability of wood anatomical parameters (**Figures 5**, **7**). Besides showing great potential in accurately reconstructing wood density at high resolutions (Björklund et al., 2019), the method is also capable of correcting for tilted vessel due to the presence of 3D information. Its rarer application is due to the need for expensive equipment and specialized analysis software. An additional downside of this method is that it requires data acquisition and reconstruction times as the resolution increases (Jacquin et al., 2017), yet very low labour cost as it has a high automation potential. It is due to this reason that we selected our target resolution (0.28 pixel per μm), even though that the resolution can be increased to also include the fibre dimensions (Van den Bulcke et al., 2009; Dierickx et al., 2024). Yet, the potential use for micro-CT has been proven an effective tool in obtaining a multitude of wood anatomical characteristics (Steppe et al., 2004; Mayo et al., 2010). Micro-CT allows us to obtain wood density profiles which can be directly compared to the wood anatomical features (De Mil et al., 2016). The new workflow, as described in Lehnebach et al. (2021), could for example be a critical new technique in estimating biomass increments within the tree ring for any tree species. This method also allows for the detection of wood anatomical features in 3D, permitting the correction of changing angles of the vessels and fibres (Trtik et al., 2007; Brodersen, 2013). Finally, micro-CT can be applied to tropical hardwood species, for which normally it would be challenging to use the thin-section method, due to difficulties in cutting the sample (i.e., Van Camp et al., 2018; Tarelkin et al., 2019), allowing micro-CT to boost tree ring research (Van den Bulcke et al., 2019). The development of high-resolution X-ray computed tomography is thus recognized by the scientific community as a critical and non-destructive technical advancement in QWA (Gennaretti et al., 2022) and our results should further motivate its implementation within QWA research, especially given the continuous progress in acquisition and reconstruction speed.

### Concluding remarks and ways forward

Our systematic uncertainty analysis of the three most commonly applied image acquisition methods in analysing QWA time-series reveals their potential in effectively extracting wood anatomical parameters. Particularly, hydraulic parameters related to cell number and lumen dimensions appear easily extractable with the surface picture method, making it possible to process large quantities of samples in a relatively short time compared to wood anatomical thin sections. However, it is not advised to apply such a method on species with a maximum lumen area below 4000 μm², even with comparably high resolution of 6000 dpi used in this study (see **Figure 2** with *A. glutinosa*), or on species with unclear visual ring boundaries. We also presented an effective way to assess within-tree radial profiles with all methods. Since the temporal dynamics of angiosperm wood formation are still under early exploration (Balzano et al., 2018; Noyer et al., 2023), the presented results provide a first benchmark on the accuracy of these different methods for angiosperm species and should motivate their combined application in QWA, especially when using the sectorial approach.

From our results, we estimate that it is possible to compile wood anatomical parameters collected with various methods, as long as the maximum vessel size is above 4000 μm^2^, paving the way forward to create a generalized database which includes these measurements for large-scale spatial analyses, similar to existing tree ring databases (e.g., Babst et al., 2017; Babst et al., 2019; Klesse et al., 2023). However, a clear issue from our results is that species with larger vessels, like ring-porous species, tend to be sensitive in their wood anatomical parameters to the number of vessels considered in the calculation. This sensitivity is highly dependent upon the tangential width of the sample. A systematic assessment of this effect on the climate sensitivity would be helpful in establishing the exact width needed to obtain robust climatic signals. Also, our uncertainty assessment can be expanded by creating QWA chronologies from each of the methods to show how climatic responses are impacted, as demonstrated in the comparison by Björklund et al. (2019). This would however require higher sample replication and a higher and standardized number of tree rings per sample than in our study. With the sectorial approach presented here, it is possible to move beyond isolating solely the first row of vessels (Souto-Herrero et al., 2017) and delve into more detail on the radial pattern changes of QWA parameters and inspect their individual climate sensitivities. These investigations could be of great value as they would provide even more robust recommendations for method selection and their specific use in the QWA community (von Arx et al., 2021).

## Data Availability

The raw data supporting the conclusions of this article will be made available by the authors, without undue reservation.
