# Supplementary material for: Quantitative vessel mapping on increment cores: a critical comparison of image acquisition methods
Source: Front Plant Sci. 2025 Feb 12;16:1502237. doi: 10.3389/fpls.2025.1502237 (PMC11863283; doi:10.3389/fpls.2025.1502237)
Supplement: Supplementary file 1 [file DataSheet1.docx]

**Title:**

Quantitative vessel mapping on increment cores: A critical comparison of image acquisition methods

**Authors:**

Richard L. Peters^1,2,3*^, Stefan Klesse^4,5^, Jan Van den Bulcke^6,7^, Lisa M.Y. Jourdain^4,5^, Georg von Arx^4,5^, Alba Anadon-Rosell^8,9^, Jan Krejza^10,11^, Ansgar Kahmen^2^, Marina Fonti^4^, Angela Luisa Prendin^12^, Flurin Babst^13,14^, Tom De Mil^3^

**Affiliations:**

^1^Tree Growth and Wood Physiology, TUM School of Life Sciences, Technical University of Munich, Hans-Carl-v.-Carlowitz-Platz 2, 85354 Freising, Germany

^2^Department of Environmental Sciences – Botany, University of Basel, Schönbeinstrasse 6, CH-4056 Basel, Switzerland

^3^Forest Is Life, TERRA Teaching and Research Centre, Gembloux Agro Bio-Tech, University of Liège, Gembloux, Belgium

^4^Swiss Federal Institute for Forest, Snow and Landscape Research WSL, 8903 Birmensdorf, Switzerland

^5^Oeschger Centre for Climate Change Research, University of Bern, Bern, Switzerland

^6^Laboratory of Wood Technology (UGent-Woodlab), Department of Environment, Faculty of Bioscience Engineering, Ghent University, Ghent, Belgium

^7^UGCT - UGent Centre for X-ray Tomography, Ghent University, Ghent, Belgium

^8^CREAF, 08193 Cerdanyola del Vallès, Catalonia, Spain

^9^Institute of Botany and Landscape Ecology, University of Greifswald, Greifswald, Germany

^10^Global Change Research Institute of the Czech Academy of Sciences (CzechGlobe), Bělidla 986/4a, 603 00 Brno, Czech Republic

^11^Department of Forest Ecology, Faculty of Forestry and Wood Technology, Mendel University in Brno, Zemědělská 3, 613 00 Brno, Czech Republic

^12^Department of Land, Environment, Agriculture and Forestry, University of Padua, Viale dell'Università 16 - 35020 Legnaro (PD) Italy

^13^School of Natural Resources and the Environment, The University of Arizona, Tucson, USA

^14^Laboratory of Tree-Ring Research, The University of Arizona, Tucson, USA

*Corresponding author = richard.peters@tum.de

RLP: <https://orcid.org/0000-0002-7441-1297>

SK: <https://orcid.org/0000-0003-1569-1724>

JVdB: <https://orcid.org/0000-0003-2939-5408>

LMYJ: <https://orcid.org/0009-0008-9218-1262>

GvA: <https://orcid.org/0000-0002-8566-4599>

AAR: <https://orcid.org/0000-0002-9447-7795>

JK: <https://orcid.org/0000-0003-2475-2111>

AK: <https://orcid.org/0000-0002-7823-5163>

MF: <https://orcid.org/0000-0002-2415-8019>

ALP: <https://orcid.org/0000-0002-5809-7314>

FB: <https://orcid.org/0000-0003-4106-7087>

TDM: <https://orcid.org/0000-0001-6207-9613>

**Supporting information:**

**Table S1.** Overview of data from unpublished and published wood anatomical sites.

| **Site (Country)** | **Latitude (°N)** | **Longitude (°E)** | **Species** | **Trees (number)** | **Sources** |
| --- | --- | --- | --- | --- | --- |
| Hofstetten forest (CH) | 47.4686 | 7.5024 | *Carpinus betulus* | 4 | Peters et al. 2023 |
|  |  |  | *Fagus sylvatica* | 4 |  |
|  |  |  | *Larix decidua* | 4 |  |
|  |  |  | *Picea abies* | 4 |  |
|  |  |  | *Pinus sylvestris* | 4 |  |
|  |  |  | *Quercus petraea* | 4 |  |
| Lötschental elevational transect (CH) | 46.3934 | 7.7639 | *Picea abies* | 10 | Peters *et al.* 2021 |
| Rhone valley near Gampel (CH) | 46.3026 | 7.7411 | *Picea abies* | 3 | *unpublished* |
| San Vito di Cadore (IT) | 46.4501 | 12.2162 | *Picea abies* | 2 | *unpublished* |
|  |  |  | *Pinus sylvestris* | 2 | *unpublished* |
| Soroe, ICOS flux tower site (DK) | 55.4869 | 11.6458 | *Fagus sylvatica* |  | Peters *et al.* 2020 |
| Lanžhot (CZ) | 48.6815 | 16.9465 | *Quercus robur* | 7 | *unpublished* |
| Bílý Kříž (CZ) | 49.0359 | 17.9698 | *Picea abies* | 8 | *unpublished* |
| Rájec (CZ) | 49.4437 | 16.6965 | *Picea abies* | 7 | *unpublished* |
| Eldena natural protection site (DE) | 54.0793 | 13.4762 | *Quercus petraea* | 3 | Resente et al. 2021 |
|  |  |  | *Fagus sylvatica* | 1 |  |
| Central Siberia (RU) | 61.2500 | 89.6333 | *Betula pubescens* | 1 | *unpublished* |
| Zurich (CH) | 47.3600 | 8.4530 | *Fraxinus excelsior* | 72 | Klesse et al. 2021 |
| Hünenberg (CH) | 47.1659 | 8.4163 | *Fraxinus excelsior* | 21 | *unpublished* |
| Wöpkendorf, peatland forests  (DE) | 54.1347 | 12.5127 | *Alnus glutinosa* | 1 | Anadon-Rosell et al. 2022 |


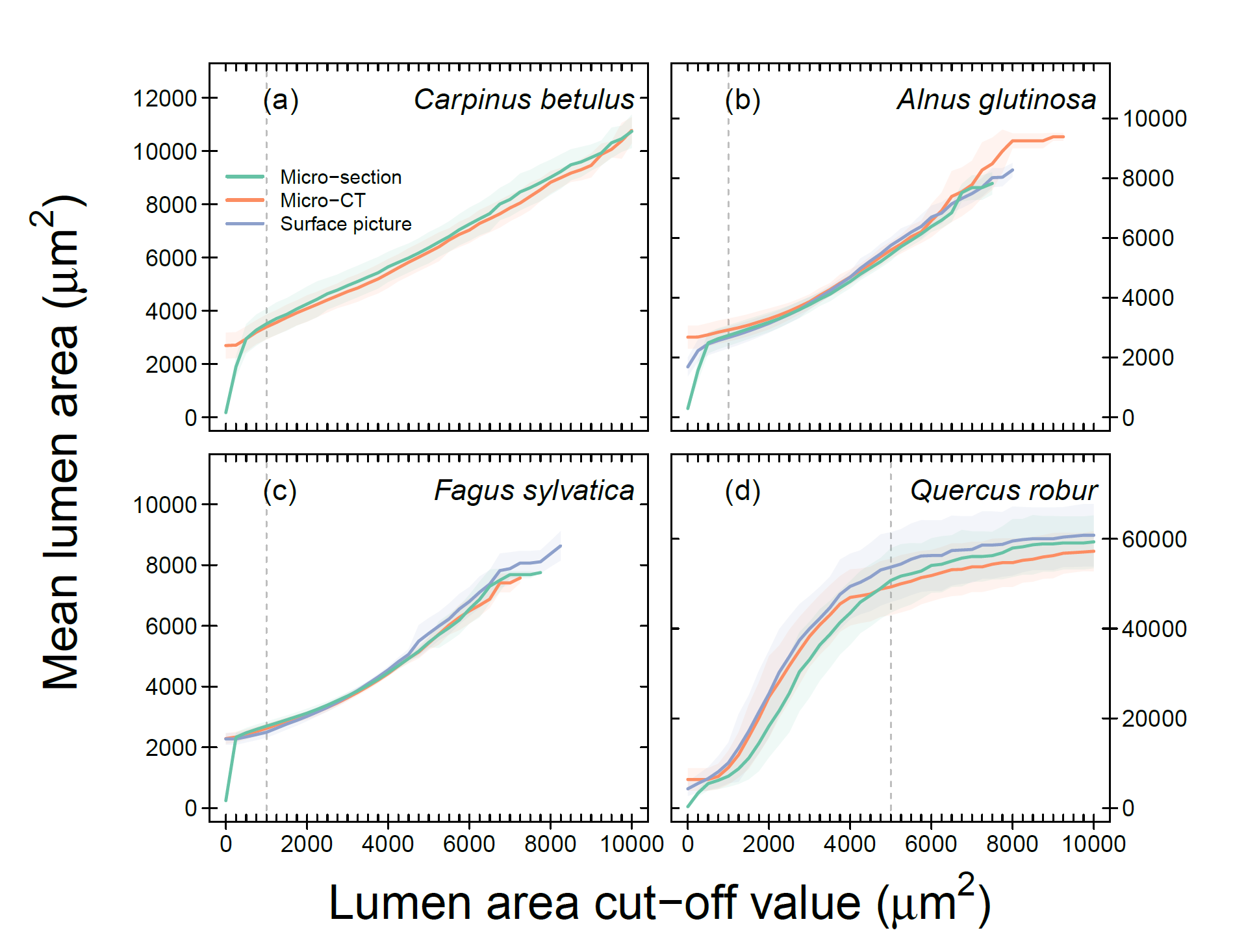


**Figure S1.** Impact of variable cut-off values of the cell lumen area and its effect on the mean lumen area of the tree ring. For all four species, *Carpinus betulus* (a), *Alnus glutinosa* (b), *Fagus sylvatica* (c), and *Quercus robur* (d), the mean trajectory is provided for the impact of the cut-off value for the mean lumen area of all rings considered for the species (see Table 1). The shading around the mean line indicates the standard deviation. Each image acquisition method is indicated with a colour. Cut-off values were selected based on the first point at which the methods were highly comparable in mean lumen area.


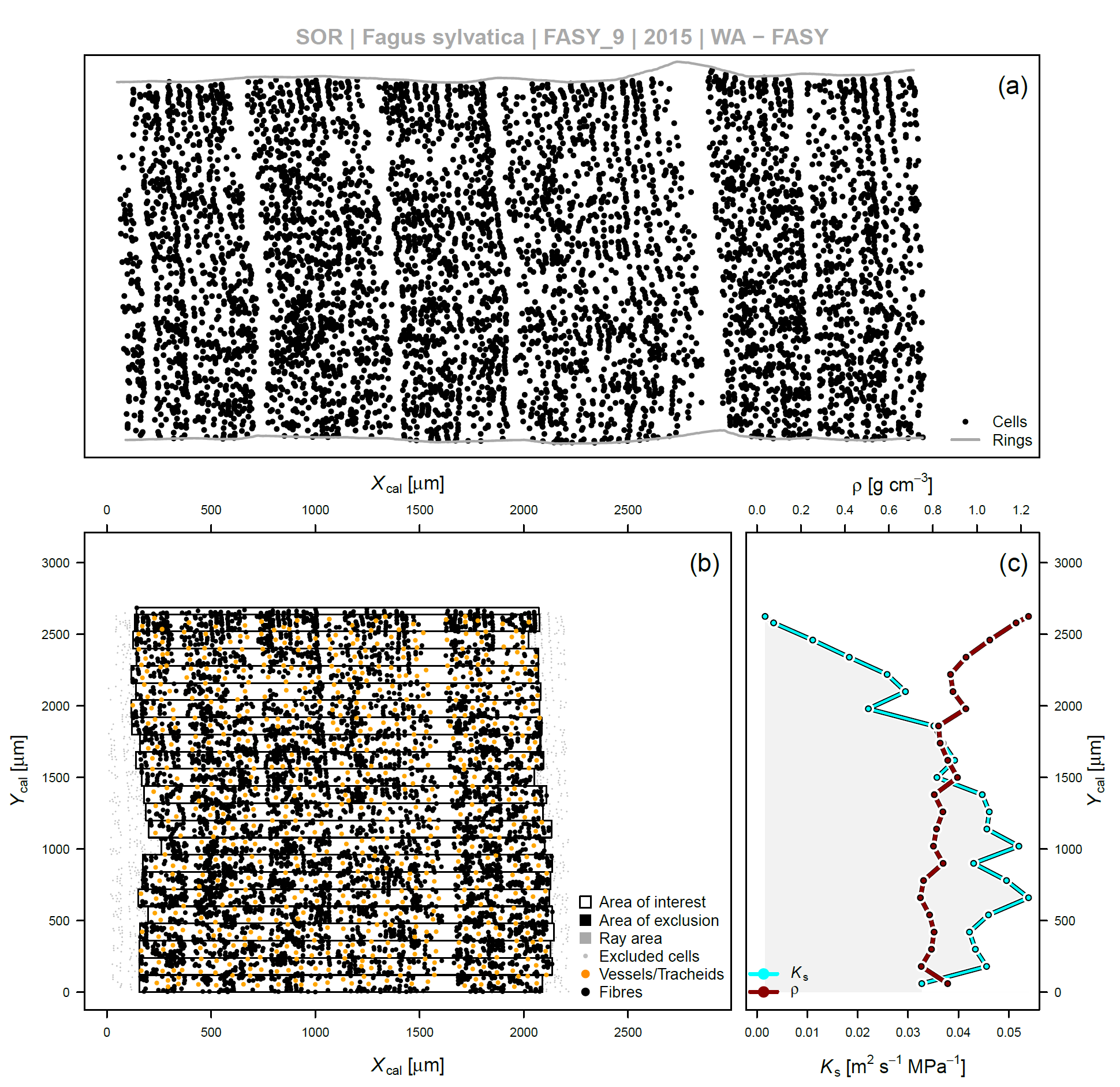


**Figure S2.** Example of intra-annual quantitative wood anatomical (QWA) data processing, for the 2015 tree ring obtained from a Fagus sylvatica tree (FASY24; Table 1). (a) Relative centre position of vessels and fibres detected by the image analyses software (output from ROXAS). (b) Application of a sectorial binning approach relative to the ring boundary according to the relative position (*Y*_cal_ and *X*_cal_). Detection algorithms are included which detect rays and areas of exclusion within the bin area (or area of interest). (c) Average theoretical hydraulic conductivity (*K*_s_) and wood density (*ρ*) per bin, as defined in (b). Within this example each bin is 120 μm in length.

**
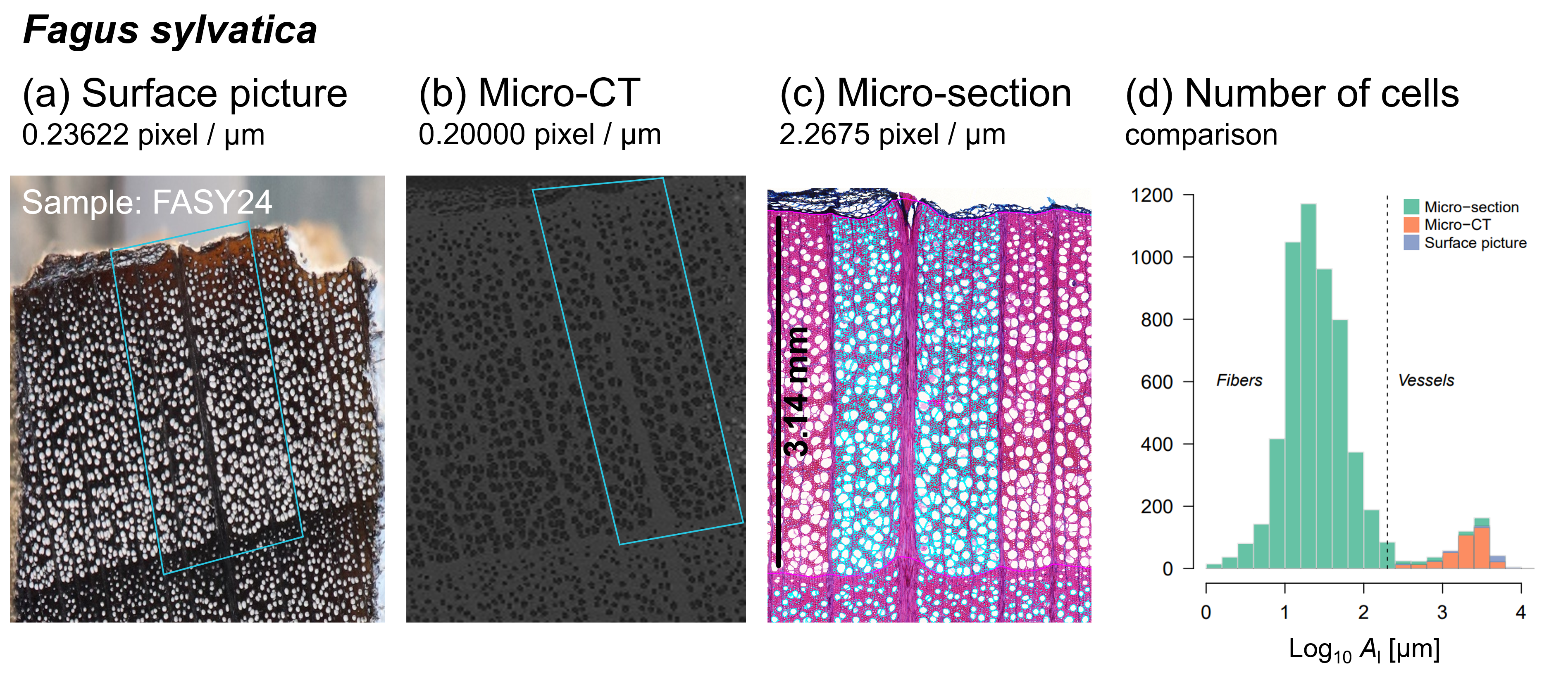
**

**Figure S3.** Overview of the image acquisition methods. The surface picture (a), micro-CT (b), and a micro-section (c) are shown from a *Fagus sylvatica* tree for the year 2017 (FASY24; Table 1). From a selected area a histogram is provided with the log transformed frequency distribution of fibres and vessels according to the different methods (d).

**
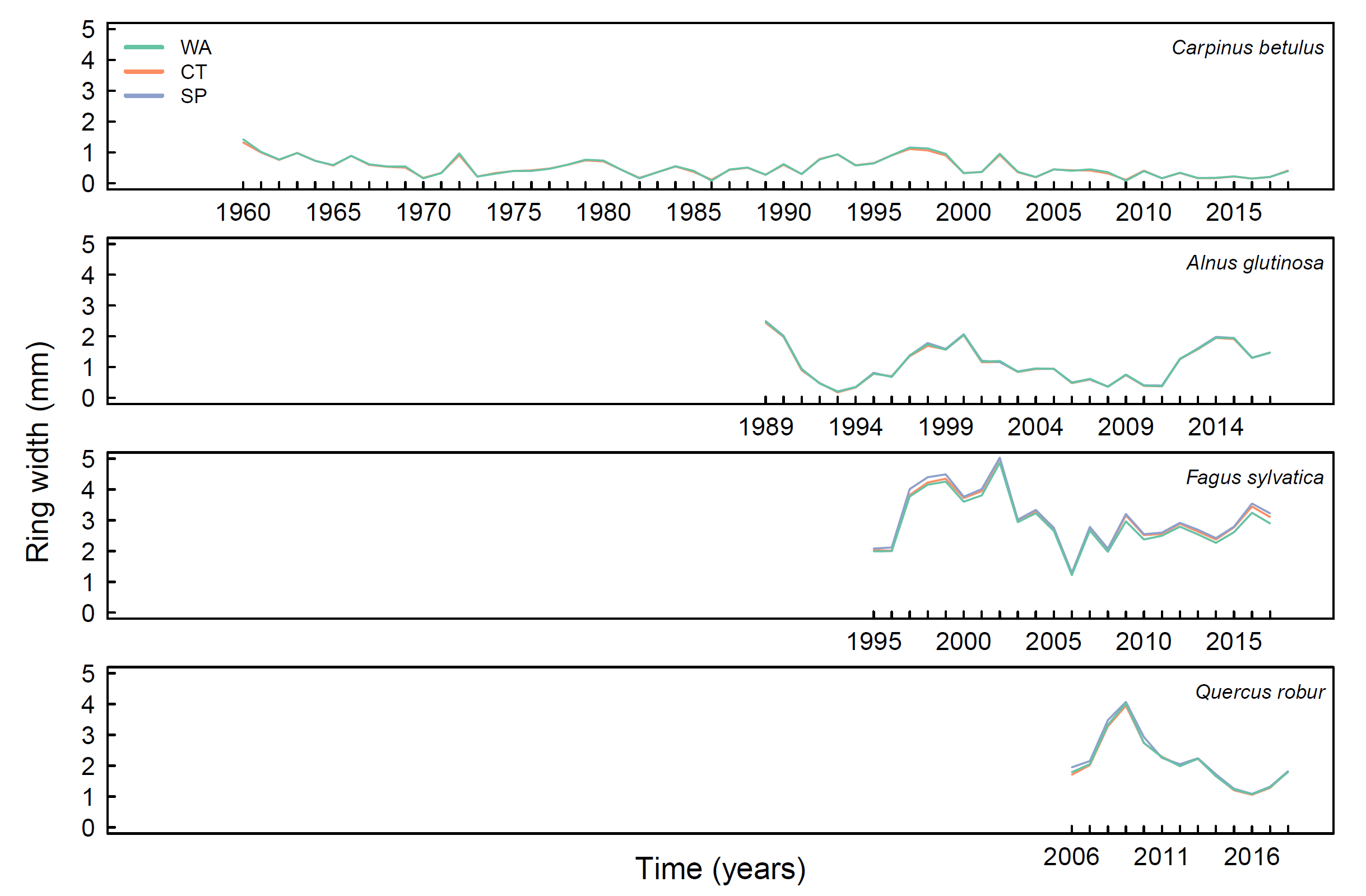
**

**Figure S4.** Ring-width measurements for all target species and image acquisition methods. Each species is indicated with a specific column moving from diffuse-porous (top) to more ring-porous (bottom). The colours indicate the image acquisition methods, namely, WA = thin section, CT = Micro-CT X-ray tomography, and SP = surface picture.


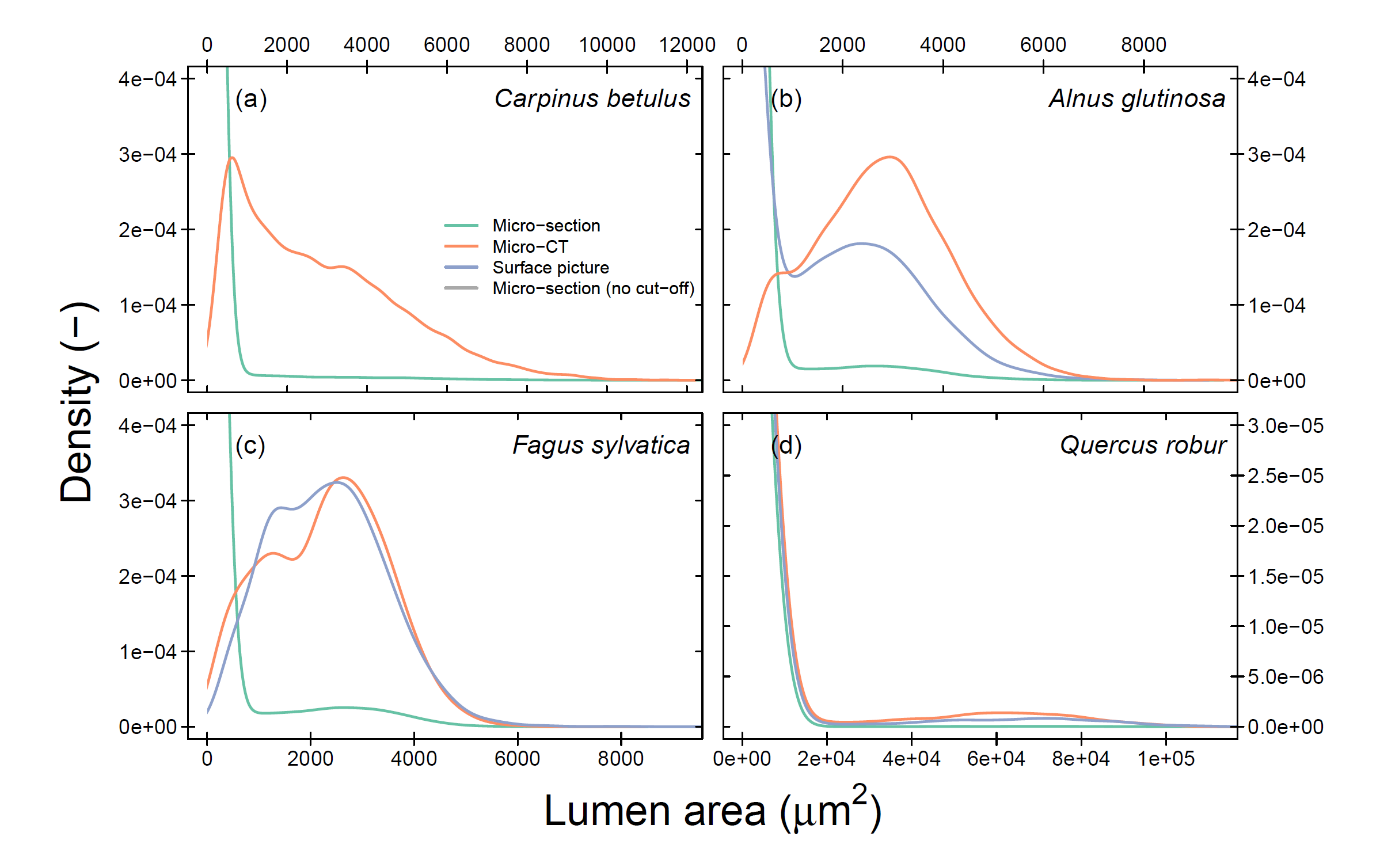


**Figure S5.** Density plot of the cell-specific lumen area distribution across all tree rings per species. Colours indicate the different image acquisition methods. The smoothed density plot is presented for the species *Carpinus betulus* (a), *Alnus glutinosa* (b), *Fagus sylvatica* (c), and *Quercus robur* (d).
